# Therapy Outcome of a T-Cell-Rich-B-Cell Lymphoma (TCRBCL) Patient with R-CHOP in Ibadan, Nigeria: a Case Report.

**DOI:** 10.4084/MJHID.2011.008

**Published:** 2011-03-16

**Authors:** J.A Olaniyi, A.O. Oluwasola, A Ibijola

**Affiliations:** 1 Department of Haematology and; 2 Histopathology, University College Hospital, Ibadan, Nigeria

## Abstract

T-cell-rich B-cell lymphoma (TCRBCL) is considered a rare variant of aggressive B cell lymphoma characterized by few neoplastic B cells and a large reactive infiltrate with striking similarities to nodular lymphocyte predominant Hodgkin’s lymphoma.

A case of a 46 year old man referred with a 5 months history of generalized lymphadenopathy, weight loss, low grade pyrexia and two separately reported lymph node histology consistent with TCRBCL is described.

The clinical course was indeed aggressive because in spite of initial treatment with four cycles of CHOP combination chemotherapy, followed by R+CHOP(x 6 cycles), signs of tumor re-growth/infiltration were frequently observed. Also, recurrent infection was frequent, troublesome and eventually became overwhelming resulting to the loss of the patient.

This case, being the first case of TCRBCL diagnosed by immunohistochemical confirmation and managed at this centre with R-CHOP, is presented to highlight the dilemma in making diagnosis, clinical challenges faced and rituximab therapy outcome especially in resource poor country. It will also serve to increase our index of suspicion and the need reinforce immunohistochemistry in the diagnosis of lymphoma.

## Introduction:

T-cell Rich B-Cell Lymphoma (TCRBCL), previously considered to be an extrapulmonary form of Lymphomatous Granulomatosus. It is generally considered to be a B cell lymphoma with an exuberant benign T cell reaction.^1^,^2^ It was first described as a clinicopathological entity in 1972.^3^ It’s diagnosis is based on histological triad of: polymorphic lymphoid infiltrate composed of small lymphocytes, plasma cells and variable number of large atypical mononuclear cells; angitis due to transmural infiltration of arteries and veins by lymphocytes (a process quite different from vasculitis in which acute and chronic inflammatory cells are found with associated cell wall necrosis) and granulomatosis (central necrosis within the lymphoid nodules and not granuloma formation).^4^ Therefore, by 1990, the disease was viewed as an extranodal, angiocentric, T cell lymphoma with predilection for the lungs.^5^

Flow cytometric immunophenotyping and polymerase chain reaction that determine T-cell receptor and immunoglobulin clonality is the hallmark of diagnosis.^6^ It is a rare subtype of lymphoma, with distinctive histology and immunophenotype. Histologically it is composed of, scattered atypical large neoplastic cells in a background of small lymphocytes and sometimes histiocytes. The large cells exhibited CD20+, CD79a+, EMA+, CD15- and CD30-phenotype. On the other hand, the background small lymphocytes were CD3 and CD45RO-positive. Most of these background T cells express CD8 and TIA-1, while they are mostly CD57-negative. The histiocytic cells are CD68-positive;. In-situ hybridization for EBER 1/2 show negative nuclear signals. Immunoglobulin heavy chain gene rearrangement study usually reveal clonal pattern.

The above features are helpful in delineating this entity from Nodular Lymphocyte predominant, Hodgkin lymphoma(NLPHL), reactive lymphoid hyperplasia and lymphomatoid granulomatous.

Although LBCL lymphoma is the commonest type of Non-Hodgkins lymphoma (NHL) in Ibadan, Nigeria; diagnosis of TCRBCL is rare and has not being reported in our sub region.

## Case Presentation:

The patient was a 46 year old man first seen in February 2009 with a referral from a Private Facility in Lagos state. He presented with a 5 month history of generalized lymphadenopathy, weight loss and low grade pyrexia. Essential findings on physical examination included bilateral axillaries’, inguinal, submandibular lymphadenopathy, size ranging from 2x2cm to 2x6cm; bilateral paedal oedema up to the groin, including scrotal and penile oedema, and non- tender generalized sub-cutaneous nodules. Examination of other systems was normal.

**Figure 1:**
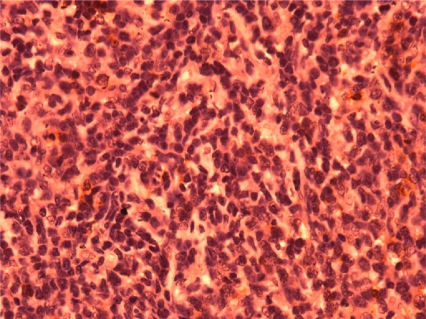
Photomicrograph showing atypical large lymphoid cells in a background containing normal small lymphocytes.

Preliminary report of FNAC received by the Institution histopathologist was reactive follicular hyperplasia. But subsequently two biopsy, one of subcutaneous nodule and the other of an inguinal lymph node were performed and the histology pattern of both biopsy was consistent with Non-Hodgkin’s Lymphoma of T-cell rich diffuse large B-cell lymphoma (Figure 1) as certified by two independent pathologists. This diagnosis was quently subseconfirmed by immunohistochemistry showing reactive cells were positive for CD3, CD5 and Bcl2 while the neoplastic cells component were CD20 positive; CD10 and cyclin D1 was negative. All the same, a third opinion was sought from the institution’s histopathologist, the histology report came much later (because of logistic reasons) as Hodgkin’s Disease, Mixed Cellularity sub-type. As at that time of receiving this third opinion report, treatment had being commenced because of the poor state of the patient and patient had shown very good response to 4 cycles of CHOP(Cyclophosphamide Adriamycin, Oncovin and prednisolone) chemotherapy.

Further investigations included Abdominal Ultrasound which reported para-aortic and para-iliac lymphadenopathy, chest X-ray showed bilateral streaky opacities in both lung fields suggestive of Pulmonary tuberculosis and bone marrow aspiration showed significant (about 30%) abnormal lymphoid infiltration. He had normal biochemical findings; retroviral screening and VDRL were negative

After well tolerated 4 cycles of CHOP, there was a total regression of the enlarged lymph nodes and other accompanying signs. Also, FBC and biochemical investigations were within normal. At this juncture, chemotherapy was suspended for about 3 months and the waiting period was used to source for Rituximab. At the tail end of the third month, he had evidence of left lobar pneumonia and features of disease progression vis a vis recurrence of lymph nodes, reappearance of skin nodules at the trunk and elevated liver enzymes. The pneumonia responded to combination of antibiotics. When he subsequently commenced 21 day cycle of R-CHOP, of which he had six cycles, there was impressive evidence of disease remission.

However, patient developed right cervical lymphadenitis and abscess collection after the 5^th^ cycle of R-CHOP. This was drained and the aspirate which was sent for culture yielded Klebsiella, sensitive to Levofloxacin and which the patient had for 3 weeks. Three weeks after the resolution of the right cervical abscess he developed peri-anal abscess which had to be drained by the surgeons. Retroviral screening was negative but HBsAg was positive. He also developed numbness of both feet with progressive inability to walk. This was initially thought to be induced by vincristine.

While awaiting Neurologists review in respect of his inability to walk on outpatient basis, he was brought acutely ill with greenish tinge jaundice, inability to walk, recurrence of ishiorectal abscess, distended abdomen with soft tender hepatomegaly of 12cm and soft tender splenomegaly of 8 cm. While LFT was suggestive of obstructive jaundice; abdominal USS confirmed splenic abscess and presence of hepatomegaly. The result of CT – Abdomen showed evidence of para-spinal infiltration.

He was commenced on I.V. antibiotics (Ceftrazone and Flagyl) while exploring the possibility of draining the abscess. He later developed haematochezia possibly due to acquired coagulation failure. He was supported with blood and blood products. Patient succumbed to the disease at this point.

## Discussion:

Our experience with treating lymphoma with R-CHOP is highly limited and in fact this index patient being reported happened to be the second; partly because the cost of rituximab is out of rich for an average patient in Nigeria and more so because immunohistochemistry has not fully taken its root in the country. This dismal deterioration in clinical outcome of this patient at the terminal phase gave a cause for concern especially when the best available has been offered for the patient.

TCRBCL is said to be an uncommon morphologic variant of diffuse large B Cell Lymphoma in the REAL classification and the latest WHO classification.^8^ It is characterized histopathologically by less than 10% malignant B cells amid a majority population of reactive T-lymphocytes and histiocytes.^8^ This makes the diagnosis of TCRBCL to be occasionally difficult in that it is a close differential diagnosis of other lymphoid malignancies such as Hodgkins lymphoma especially Nodular Lymphocyte Predominant HD(NLPHD) and classic HD (cHD), pleomorphic peripheral T-cell lymphoma and psudolymphoma.^9^,^10^ In working towards making a diagnosis in this patient we encountered similar problem in that after we had received 2 histological diagnoses from referral centre, we sought a third opinion of our home pathologist who in fact returned a diagnosis of HD mixed cellularity. Therapy for TCRLBCL with CHOP was however continued because there was already a good improvement to the treatment. Apart from HD another very close differential diagnosis is Peripheral T cell lymphoma. Therefore there is a great need for accurate diagnosis of this disease through careful use of immunohistochemistry. A specialized private laboratory came to our aid in this respect.

TCRBCL or T/HRBCL is an uncommon variant of DLBCL^4^ and it represents 1–3% of all DLBCL in recent series.^5^,^6^,^8^ In many case series report published;^9^,^11^,^13^ clinical presentation in advanced stage ranged from 53–91% while frequency of splenic involvement ranged from 21–60%; frequency of liver involvement and that of bone marrow involvement ranged from 4–40%. Hepatosplenomegaly occurred at the terminal phase of this patents disease while evidence of bone marrow infiltration was detected at diagnosis.

The reported mean age of occurrence of this disease is 40 years,^8^,^10^,^14^,^15^,^16^ our index patient, 40 year old male, fits into documented mean age at diagnosis compared to 5^th^ decade of occurrence for DLBCL.

TRRBCL is recognized to have both nodal and extra-nodal presentation, known to be an aggressive lymphoma that often presents as stage IV disease and with frequent bone marrow involvement.^9^ This patient also presented in the late stage i.e. Ann arbor stage IV with evidence of bone marrow involvement, clinical and radiological evidence of pulmonary infiltration; even though the liver and the spleen were not involved initially but at the terminal stage both the liver and the spleen were involved. There was pronounced weight loss and clinical evidence of pronounced para-spinal infiltration which was later confirmed by CT scan.

There is a cause for concern in this patient who had established diagnosis of TCRBCL and was able to afford CHOP chemotherapy plus rituximab but terminally succumbed to the disease with evidence of multiple organ infiltration, overwhelming sepsis and features of acquired coagulation failure.

This case report vividly highlights the difficulties of accurate diagnosis and therapy of TCRBCL in developing countries and also showed the probability of high risk of infectious complications by modern immunotherapy performed under conditions of limited resources. There is therefore the need for a high index of suspicion, adequate infectious disease work-up and provision of comprehensive immunohistochemistry facility for definitive diagnosis of this rare form of lymphoma. This will ensure prompt targeted therapy and improved chance of survival of this category of patients.
